# A Mars Environment Chamber Coupled with Multiple In Situ Spectral Sensors for Mars Exploration

**DOI:** 10.3390/s21072519

**Published:** 2021-04-04

**Authors:** Zhongchen Wu, Zongcheng Ling, Jiang Zhang, Xiaohui Fu, Changqing Liu, Yanqing Xin, Bo Li, Le Qiao

**Affiliations:** Shandong Key Laboratory of Optical Astronomy and Solar-Terrestrial Environment, School of Space Science and Physics, Institute of Space Sciences, Shandong University, Weihai 264209, China; z.c.wu@sdu.edu.cn (Z.W.); zhang_jiang@sdu.edu.cn (J.Z.); fuxh@sdu.edu.cn (X.F.); liucq@mail.sdu.edu.cn (C.L.); yqxin@sdu.edu.cn (Y.X.); libralibo@sdu.edu.cn (B.L.); leqiao@sdu.edu.cn (L.Q.)

**Keywords:** Mars simulation, Mars chamber, space instrumentation, Raman, LIBS, CO_2_ glow discharge

## Abstract

Laboratory simulation is the only feasible way to achieve Martian environmental conditions on Earth, establishing a key link between the laboratory and Mars exploration. The mineral phases of some Martian surface materials (especially hydrated minerals), as well as their spectral features, are closely related to environmental conditions. Therefore, Martian environment simulation is necessary for Martian mineral detection and analysis. A Mars environment chamber (MEC) coupled with multiple in situ spectral sensors (VIS (visible)-NIR (near-infrared) reflectance spectroscopy, Raman spectroscopy, laser-induced breakdown spectroscopy (LIBS), and UV-VIS emission spectroscopy) was developed at Shandong University at Weihai, China. This MEC is a comprehensive research platform for Martian environmental parameter simulation, regulation, and spectral data collection. Here, the structure, function and performance of the MEC and the coupled spectral sensors were systematically investigated. The spectral characteristics of some geological samples were recorded and the effect of environmental parameter variations (such as gas pressure and temperature) on the spectral features were also acquired by using the in situ spectral sensors under various simulated Martian conditions. CO_2_ glow discharge plasma was generated and its emission spectra were assigned. The MEC and its tested functional units worked well with good accuracy and repeatability. China is implementing its first Mars mission (Tianwen-1), which was launched on 23 July 2020 and successfully entered into a Mars orbit on 10 February 2021. Many preparatory works such as spectral databases and prediction model building are currently underway using MECs, which will help us build a solid foundation for real Martian spectral data analysis and interpretation.

## 1. Introduction

The environmental conditions of the Martian surface, such as the atmospheric composition, gas pressure, temperature, humidity and UV radiation levels, differ sharply from Earth ambient conditions. The mineral phases of some Martian minerals and their spectral features are closely related to environmental conditions [1,2,3]. For example, some hydrated minerals will obtain or lose some degree of structural water, and the Raman peak of water also shifts or deforms according to the low temperature [1]. On the other hand, the environmental conditions also significantly impact certain measurement techniques. Laser-induced breakdown spectroscopy (LIBS) is a typical dynamic measuring technology whose spectral profile depends heavily on the gas species and gas pressure around the target [4,5]. Therefore, environmental simulation is critical for Martian mineral detection and analysis; it is also the only feasible way to mimic the conditions of the Red Planet in the laboratory and enable the interpretation of real Martian data. Accordingly, the ChemCam team and SuperCam team calibrated and recalibrated their LIBS prediction model before/during the mission under simulated Mars conditions [6,7,8,9]. In fact, due to their irreplaceable role, planetary simulation chambers have experienced major improvements in terms of methodological models and instrumental designs, enabling various simulated experiments related to Mars [10,11,12,13], the Moon [14,15] and asteroid/cometary/solar system small bodies [15,16] for mineral analysis [10], astrobiology [11,17], instrument calibration/materials testing [6,9], planetary exploration [18] studies, and so on.

Currently, China is implementing its first Mars mission (Tianwen-1), which consists of three subtasks (one orbiter, one lander, and one rover), and this mission was launched (Wenchang Satellite Launch Center, China) on 23 July 2020. A total of 13 payloads were selected, seven of which were installed on the rover. A LIBS system, a passive VIS (visible)-NIR (near-infrared) spectrometer, and a remote microimager (RMI) were combined to make one integrated system (MarsCoDe) for Martian surface material elemental and composition detection and analysis [19].

In this study, one Mars environment chamber (MEC) coupled with four types of in situ spectral sensors (ASD spectrometer (Analytical Spectral Devices Inc., the commercial field-portable full-range UV/Vis/NIR/SWIR spectrometers), fiber Raman spectrometer, LIBS system and UV-VIS plasma emission spectrometer) was built at Shandong University. Though this system has the ability to analyze homogeneous geological samples by hyphenated spectral methods, the main aim of this paper is to introduce the work function, performance of MEC, and every coupled spectral sensor at various environmental and work parameters. Different samples and various gas pressures were used to demonstrate the performances of Raman, LIBS, and glow discharge emission spectral units. The MEC can regulate Mars-relative environmental parameters (gas components, gas pressure, sample temperature/humidity, and UV radiation dosage) and simultaneously or sequentially execute multiple in situ spectral measurements. The spectral characteristics (VIS-NIR, Raman, and LIBS) of some geological samples can be obtained within a few seconds under given Martian environmental conditions. In this study, (1) the structure, function, and performance of MEC were systematically introduced; (2) the spectral features of some geological samples were recorded and analyzed by in situ spectral sensors under various simulated Martian conditions; (3) the CO_2_ glow discharge induced by Mars dust storms/devils were simulated in the MEC. In addition, the free radicals and the electrochemical reaction products were monitored in situ by emission spectrometry during CO_2_ glow discharge. This system establishes a pathway to link the experimental data from the laboratory to the real spectral data obtained from Mars missions.

## 2. Mars Environment chamber

### 2.1. Chamber Structure

**Figure 1:**
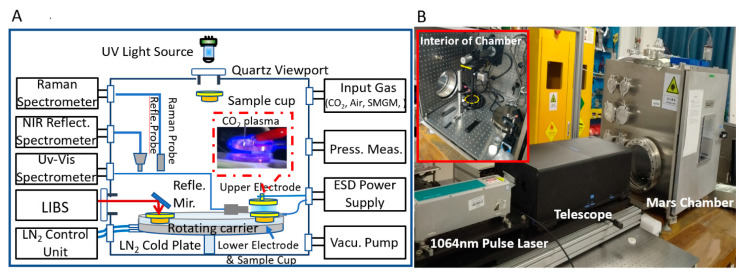
(**A**) Schematic diagram of the Mars chamber and peripheral function units. The inset is a photograph of CO_2_ plasma generated between two copper plate electrodes. (**B**) Photograph of the Mars chamber. The upright image is a photograph of the interior of the Mars chamber. The spectral sensor can be used for in situ detection of minerals in chambers or free radicals in CO_2_ plasma.

As shown in Figure 1, the Mars chamber mainly consists of a customized stainless box-shaped vacuum chamber (25 × 25 × 25 inches, Kurt J. Lesker Company, Jefferson Hills, PA, USA). A dozen flange interfaces were designed and mounted on the walls of the chamber, which were fixed by electronic/optical feedthroughs for sample spectral data collection and chamber environmental parameter control, maintenance, and monitoring. Two circular quartz viewports were mounted on the right (diameter: 200 mm) and top (diameter: 100 mm) walls of the chamber for LIBS laser input, plasma signal transmission, and UV radiation. Rectangular glass observation widows (width: 150 mm, height: 250 mm) were installed in the middle of the front door. Five optical breadboards were fixed on the inside wall of the chamber (except for the front door) for accessory fixation and adjustment.

The MEC was designed to simulate the Martian gas pressure, surface temperatures, humidity, and UV radiation. Under these simulated conditions, multiple in situ spectroscopic measurements of geological samples can be performed well.

### 2.2. Chamber Function

#### 2.2.1. The Regulation of Gas Pressure and Components

For accurate and reliable gas pressure control and measurement, a gas pressure controller (946 Vacuum System Controller, MKS Instruments, Andover, MA, USA), a pressure sensor (model: KJL300808, MKS Instruments, Andover, MA, USA), an electronic mass flow meter, and an oil-free vacuum pump (XDS35i, Edwards Ltd., Flintshire, UK) were used to dynamically control the gas pressure (from 0.1 to 1000 Pa) inside the chamber. A gas cylinder was used to supply pure CO_2_ gas or commercial Martian simulated gas mixtures to the MEC as the working gas. A customized four-channel gas mixer was also used for in situ gas mixing (CO_2_, N_2_, Ar, and O_2_) during pumping by using four electronic gas flowmeters (Flowmethod Co., Ltd., Shenzhen, China). The gas species and their relative amounts can be well regulated.

**Figure 2:**
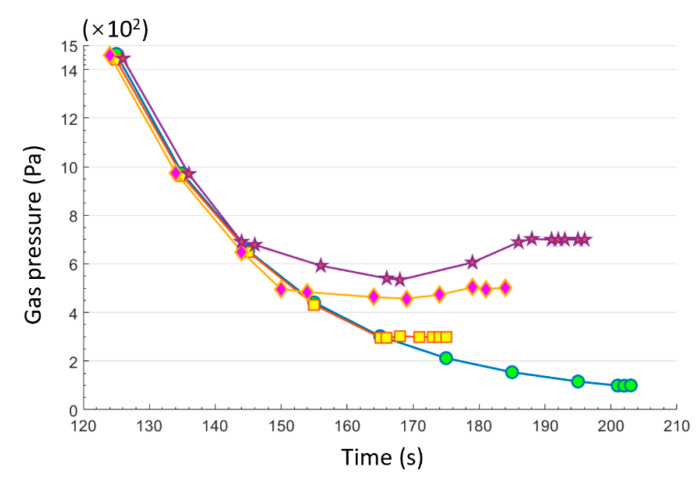
Typical curves of vacuum pressure with pump-down time.

The gas pressure control system worked effectively, with relatively high accuracy and reproducibility (±2.0 Pa at 700 Pa). The gas pressure of the chamber can be well controlled. As seen in Figure 2, the gas pressure inside the chamber continuously decreased to the set point after the gas pressure controller system began to work (pressure < 1100 Pa). The minimum pressure (100 Pa) was achieved within ~200 s. Some gas pressure curves showed slight oscillations near the set point, for example, 700 Pa in this case. This is a normal phenomenon for gas control in vacuum systems [20].

#### 2.2.2. The Regulation of Temperature and Humidity

In the chamber, only the temperature of the test samples was regulated by a customized heating and cooling stage (HCST, Instec, Inc., Boulder, CO, USA), which was installed inside the MEC and connected to a mK1000 temperature controller and a liquid nitrogen (LN_2_) pump by one electronic feedthrough and two gas feedthroughs. The diameter of the cooling area was 200 mm. LN_2_ was stored in a Dewar outside the MEC for low-temperature cooling, and this was pumped into the HCST through one gas feedthrough and pumped exhaust gas (evaporated N_2_ gas) was pumped out from the other. The coiled resistance wire was uniformly laid out inside the cooling stage for heating. The controllable temperature (−150 to 200 °C) covered the temperature range of the Martian surface with good uniformity and stability (≤±0.5 °C).

**Figure 3:**
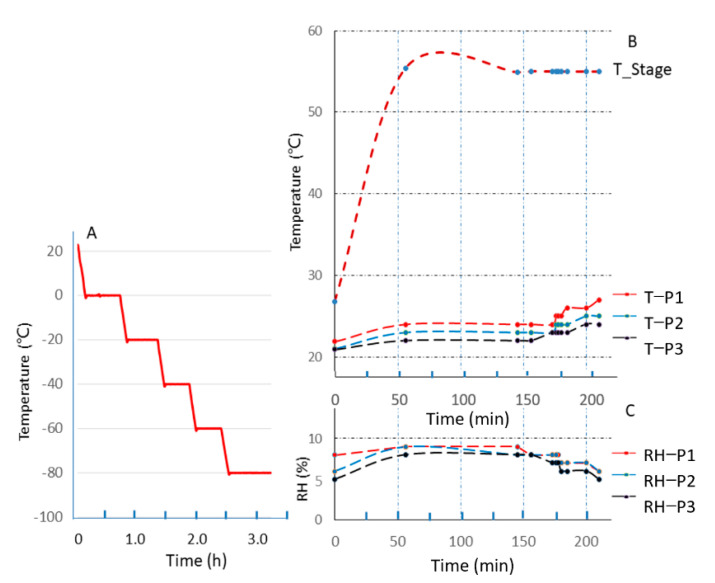
The heating and cooling stage (HCST) thermal and humidity cycling profile achieved in the chamber. (**A**) The temperature profile of HCST during cooling; (**B**) The temperature profiles of HCST and three-channel temperature humidity sensor above the HCST during heating; (**C**) The relative humidity profiles of the three-channel temperature humidity sensor above the HCST during heating.

A series of low-temperature experiments was performed from room temperature to −80 °C with an interval of −20 °C. The results are shown in Figure 3A. It took approximately half an hour for every 20 °C decrease in temperature. There were also several transient temperature oscillations at the time when the set point was first reached. The set point temperature can be well maintained for dozens of hours without human intervention.

Because of the spontaneous volatilization of water during pumping, controllable humidity is difficult to realize in a closed low-pressure chamber, which is important for the phase transition research into Martian hydrous minerals. However, it is impractical for relative humidity (RH) control by mixing different volumes of dry and wet gas because of the condensation of water vapor on the metalware housing at various temperatures in the closed chamber.

In this study, a beaker filled with 200 milliliters of pure water was placed inside the MEC near the HCST (distance: 100 mm). A three-channel temperature humidity sensor (Xiannou, Xian, China) was used for in situ monitoring of the temperature and RH values at three different heights (Probe 1: 71.4 mm; Probe 2: 93.0 mm, and Probe 3: 138.3 mm) immediately above the HCST. When the gas pressure of the MEC remained stable at 900 Pa, the temperature of the HCST was set at 55 °C, and it began to heat. The monitoring temperature and RH values with time are shown in Figure 3B.

It can be seen from Figure 3B that an equilibrium temperature of the set point can be achieved within 50 min. The temperatures of the three probes exhibited a very small temperature difference (2–3 °C) and gradient (1–2 °C) (uncertainty, ±0.5°C) due to the lower thermal conductivity at low pressure. The monitored RH of the three probes also showed a relatively stable value between 6% and 8% (uncertainty, ±0.5%), which means that this method can effectively obtain a stable RH in a narrow range during pumping. As is already known, it takes more time to reach a stable thermal equilibrium and let the heat be delivered over a long distance under low gas pressure. In this case, the HCST took about 1 h to stabilize at its setpoint. After 150 min, the heat was transmitted to the three probes above the HCST, which caused a 1–2°C increase in temperature and a decrease in relative humidity of 1–2%. This is an acceptable response for the humidity to the temperature in a closed space.

### 2.3. UV Radiation System

The Martian surface is directly exposed to a high dose of UV radiation from the Sun due to the lack of an Earth-like dense atmosphere. The daily UV (UVC and UVB: 200–315 nm) fluence on the surface of present-day Mars is 361 kJ/m^2^, which is one order of magnitude higher than that on our planet (39 kJ/m^2^) [21]. Strong UV radiation has an important effect on the evolution of matter, such as atmospheric composition [22,23], surface salts [23,24], and even organic [25] or biological materials [26], not only in the upper atmosphere but also on the Martian surface.

Xe lamps have very high radiation power in the UV band and are always used as UV radiation sources for Mars-related studies. One Xe lamp (500 W, SOFN Instruments Co., Ltd., Beijing, China) was used to provide a continuum spectral output (200–1100 nm) through a quartz window for sample radiation in our laboratory.

### 2.4. Sample Delivery System

Since multiple spectral sensors were coupled on the MEC and ready for use, a sample delivery system (inset of Figure 1B) was needed to deliver samples to the right place for spectral measurement. A rotary stepping motor was hung in the middle of the chamber by a cantilever fixed on the wall of the chamber. The sample rotating platform (SRP) was an aluminum circular disk with 16 evenly distributed circular holes near the rim and was hung in parallel and near the bottom of the MEC by using a straight bar to interlink the rotary stepping motor. The HCST can be installed under the SRP for sample heating/cooling. By this design, the SRP can expediently deliver a plurality of samples to different spectral sensors under harsh conditions. During operation, the powder samples were filled in copper cups that were placed in the holes of the SRP for cooling/heating, delivery and spectral measurement. All the signal/control wires and power cords were connected to a controller outside the chamber by using electronic feedthroughs.

## 3. Spectroscopic Sensors

The MEC was equipped with four in situ spectral sensors, i.e., ASD, fiber Raman, stand-off LIBS, and emission spectrometers, which can provide complementary spectral information for mineral analysis. The ASD, fiber Raman and emission spectrometers each have their own fiber probes, which were installed inside the MEC by optical feedthroughs. All the remaining parts were installed outside the MEC. One aim of this design is to record the spectral features of some geological samples affected by the conditions inside the chamber. The other aim is to prevent the spectrometers from interference or even harm from harsh conditions inside the chamber. The safest way of doing this is to install all the major critical components outside of the chamber, except for the fiber probe. However, if the spectral sensors were designed for extraterrestrial in situ exploration, such as the fly model of the Mars payload (e.g., SuperCam, SHERLOC (The Scanning Habitable Environments with Raman & Luminescence for Organics & Chemicals), etc.), all parts of the payload need to be put inside the chamber for calibration and testing. The fiber probes inside the chamber were mounted on their own motorized linear translation stages (MT1-Z8, Thorlabs, Inc., Newton, NJ, USA) and traveled in an axis perpendicular to the sample surfaces for focal adjustment. The whole LIBS unit was installed outside the MEC, which used a modified Cassegrain telescope to shoot the laser, returning the plasma signal to the spectrometer through the quartz viewport of the chamber and UV-enhanced fiber. These spectral sensors can be combined to analyze one sample. To explore the unknown and complicated targets on the Mars surface, the use of hyphenated technology is a necessary and advanced strategy. LIBS gives the main information about cations, and Raman/VIS-NIR analysis gives information about the vibration mode of the anionic group [27]. Obtaining information from these hyphenated spectroscopic methods on the same set of geological samples under well-controlled environmental conditions inside the MEC establishes a better way to link the results from laboratory experiments to the Martian in situ spectral data. For all spectral sensor measurements, unless otherwise specified, at least three spectral measurements were taken per sample, to verify the sampling homogeneity and spectral reproducibility.

### 3.1. ASD Probe

In this study, one ASD spectrometer (FieldSpec 4, ASD Inc., Longmont, CO, USA) coupled with a customized vacuum Y-shaped reflectance fiber probe was used for VIS-NIR reflectance spectral measurement by using a KF40 flange to seal the probe inside the MEC under harsh conditions. This design allows one end to connect to a light source (ASD tungsten halogen lamp), while the other connects to the ASD spectrometer. The spectral band of the ASD system is 350–2500 nm, with a spectral resolution of 3–8 nm and peak position accuracy of 0.5 nm, which gives direct information on crystal H_2_O/OH, free H_2_O, or adsorbed H_2_O on the surface of minerals by detecting overtone modes (~1.4 μm) and the combination H-O-H bending/stretching mode (~1.9 μm). The VIS-NIR reflectance spectra provide the most remarkable advances in our understanding of the water environment of the Martian surface.

**Figure 4:**
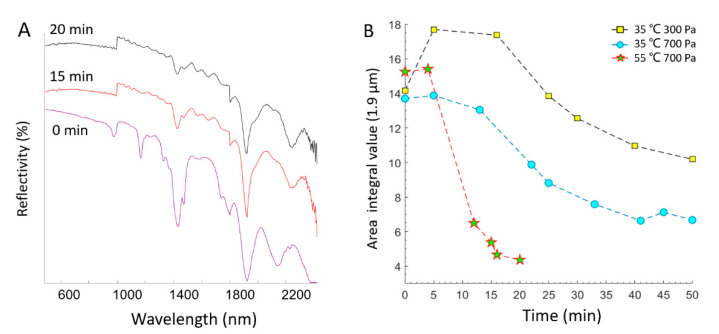
(**A**) Spectra of NaClO_4_·H_2_O during continuous monitoring in the Mars environment chamber (MEC) at 35 °C and 700 Pa. (**B**) The decrease in the absorption band area near 1.9 μm of NaClO_4_·H_2_O as a function of time in the MEC at different temperatures (35 °C, 55 °C) and gas pressures (300 Pa, 700 Pa).

The reflectance spectra of NaClO_4_·H_2_O were collected over time at 35 °C and 700 Pa with an integral time of 8.5 ms for every spectral collection (Figure 4A). In this study, the diameter of light sampling points on the samples was ~4.5 mm at a distance of 10 mm from the fiber probe to the sample surface. The H_2_O absorption depths at 1.43 and 1.93 μm exhibited a gradual reduction, which meant that an ongoing dehydration process was happening. Temperature and gas pressure are important factors for the dehydration process [1] and these were investigated with NaClO_4_·H_2_O as the starting phase in this study. As shown in Figure 4B, the absorption peak area at 1.93 μm represents the water content of NaClO_4_·XH_2_O. We found that at higher temperatures and lower gas pressures, a much larger amount of water was more quickly lost in this dehydration process. In Figure 4B, there is an unexpected increase in water absorption at the beginning of the dehydration process at 35 °C and 300 Pa because of some unknown water absorption event. Besides this, the two dehydration curves of 35 °C at 300 and 700 Pa have an almost parallel distribution along with the time. This indicates that the pressure difference of 200 Pa has little effect on the dehydration rate. However, there is a big difference in the dehydration rate when the temperature difference is 20 °C (from 35 to 55 °C). This indicates that the variation in temperature has more of an effect on the dehydration rate than the variation in gas pressure.

### 3.2. Raman Probe

An in situ fiber Raman system was made of one customized Y-shaped Raman probe (focal length: 50 mm, InPhotonics Inc., Norwood, MA, USA), an external diode-pumped solid 532 nm laser (model: 532-050-SO, 50 mW Crystal laser, Reno, NV, USA), a volume phase holographic spectrograph (Holospec-f/1.8i-Vis, Andor, Abingdon, Oxfordshire, UK), and an Andor ICCD spectrometer (DU416A-LDC-DD, Andor). Similar to the ASD probe, only the head end of the bifurcated fiber Raman probe was installed in the MEC by an optical feedthrough, whose excitation end was illuminated by a 532 nm CW (Continuous Wave) laser, and the collection end was connected to the spectrometer. Both the laser and spectrometer were installed outside the MEC. The Raman probe delivered up to ~10 mW of 532 nm excitation light at the sample surface (~400 μm in diameter at its focus (5 mm)) inside the MEC. The laser power at the Raman laser focus was measured using a laser power meter (PM100D, Thorlabs). The Andor camera attached to the spectrometer records the Raman spectra from every sample (integral time, 30 s) with a spectral resolution of ~5 cm^−1^ and peak position accuracy of ~0.1 cm^−1^ (at 520.7cm^−1^).

**Figure 5:**
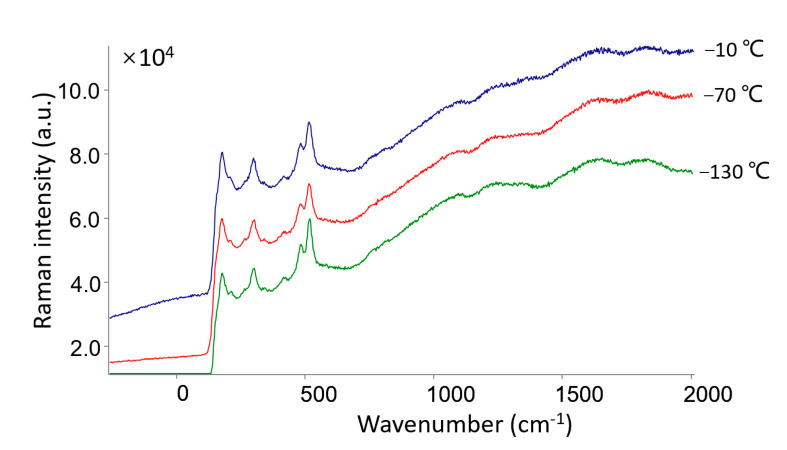
Raman spectra of feldspar under low temperatures (−10, −70, and −130 °C) at 30 Pa.

Feldspar powders (30–45 μm in grain size) were poured into an aluminum cup (diameter: 40 mm; thickness: 0.5 mm), compacted, and then placed on the HCST in the MEC. The chamber was pumped and maintained at 30 Pa. Then, the spectra of the feldspar powders were collected in situ at temperatures of −10, −70, and −130 °C when the HCST reached the set point after enough time (10 min) at thermal equilibrium. Low pressure does not change the mineral structure. Alternatively, no effect of gas pressure was found on the spectra of the rock/slice corresponding to our test. However, the porosity and stiffness of the powders might change during pumping, which could have an effect on the Raman spectra of the powder samples. This phenomenon was detected in our experiment, in which the intensity of the feldspar powder was reduced after pumping. From Figure 5, the spectral features of feldspar (i.e., peak position, intensity, and background) showed no changes at various low temperatures, even as low as −130 °C. This result indicated that low temperature does not affect the spectral characteristics and identification of anhydrous minerals on the Martian surface. Our previous study on some hydrated salts showed that the water peaks of NaClO_4_·H_2_O and Mg(ClO_4_)_2_·6H_2_O changed with decreasing temperature [1].

### 3.3. Stand-Off LIBS

The stand-off LIBS system was composed of one 1064 nm pulse laser (200 mJ, 1–20 Hz, pulse width: 8 ns, DW-200, Beamtech Optronics Co., Ltd.,Beijing, China), one modified Cassegrain telescope (main mirror diameter: 110 mm; focus length: 1.0–4.0 m), a homemade optical demultiplexer and three CCD (Charge Coupled Device) spectrometers (HR4000+, Ocean Optics, Dunedin, FL, USA) with different working spectral wavebands and spectral resolutions (UV: 240–340 nm, resolution, 0.1 nm; UV-VIS band: 340–540 nm, resolution, 0.2 nm; VIS-NIR band: 540–950 nm, resolution, 0.3 nm). The optical demultiplexer divides the incoming light into the above three wavelength bands to the individual spectrometers. Its optical structure is similar to the demultiplexer of ChemCam [28].

The peak position accuracy of this LIBS system is ~0.18 nm (at 404.6 nm). The telescope was used to shoot the pulse laser and collect the emission signal of excited plasma from the target, which was placed in the MEC under Martian conditions. A quartz fused silica viewport allowed this 1064 nm pulsed laser beam to enter the MEC, excite the target and then transmit the plasma emission spectra out to the telescope. A UV-enhanced optical fiber (core diameter: 600 μm) collected the spark of plasma from the telescope and transmitted it into an optical demultiplexer where the light was split into three bands, which were finally introduced into the three independent spectrometers. Our spectrometer (240–950 nm) covers the full spectral range of ChemCam (240–850 nm) and MarsCoDe (240–850 nm), with similar spectral resolutions.

**Figure 6:**
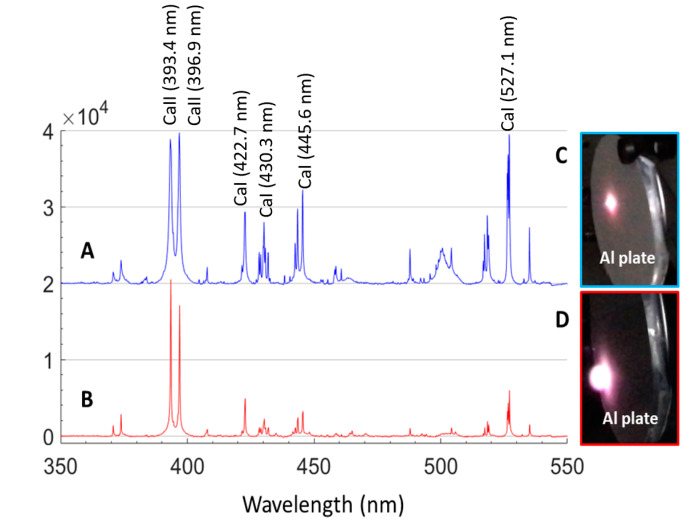
Laser-induced breakdown spectroscopy (LIBS) spectra of gypsum under Earth ambient conditions (**A**) and simulated Mars conditions (700 Pa, CO_2_ gas) (**B**); photographs of plasma excited on an aluminum plate under Earth ambient conditions (**C**) and simulated Mars conditions (700 Pa, CO_2_ gas) (**D**).

A comparative study of the mineral spectral features under different environmental conditions was performed. In this experiment, gypsum powder, CaSO_4_·2H_2_O, was pressed into a pellet and then excited by a pulse laser at 10 Hz at a distance of 2.0 m. The diameter of the sampling spot size (at focus) on the sample surface was ~200 μm at this distance. Each LIBS spectrum was averaged over three laser shots at every five different positions with an integral time of 1.0 ms. The LIBS spectra of gypsum under Earth ambient conditions (Figure 6A) and simulated Martian conditions (700 Pa in CO_2_ gas) (Figure 6B) are shown in Figure 6. The photographs of plasma excited on an aluminum plate are shown in the right part of Figure 6 (Figure 6C,D). The LIBS peaks under simulated Martian conditions have lower peak intensities, especially in spectral bands that are longer than 400 nm, have narrower spectral line widths, and have higher signal-to-noise ratios (SNRs). On the other hand, the plasma under Martian conditions (Figure 6D) was much fluffier and slightly darker than that under Earth conditions because of the lower spatial constraints at low gas pressure. The reduction in spectral line width and intensity is due to the lower collision broadening because of the low collision probability [4,5] and the luminance reduction of plasma at low gas pressure. The intensity of all the LIBS lines tended to decrease with increasing laser ablation depth. Sulfur, a typical nonmetal, is relatively harder to excite; the emission line of sulfur was not recorded in our LIBS spectra. The emission lines of oxygen at ~777.2 and ~844.6 nm were well recorded. However, those peaks were difficult to assign because the CO_2_ and O_2_ in the (simulated) Martian atmosphere interfered with those signals. Those peaks are not shown in Figure 6. Now, our main work is underway to build a LIBS database of calibration standards to prepare for analyzing the in situ LIBS data of MarsCoDe.

### 3.4. Emission Spectroscopy and Dust Event Glow Discharge Simulation Unit

Dust-event-related electrostatic discharge (ESD) has been speculated upon [29] and simulated/verified [30,31,32] under Mars-like conditions. Reactive species in these discharges, such CO_2_^+^, H^+^, OH^−^, and O, were detected in CO_2_ ESD reactions [32], which is a potentially important mechanism for the chemical evolution of Martian surface materials.

**Figure 7:**
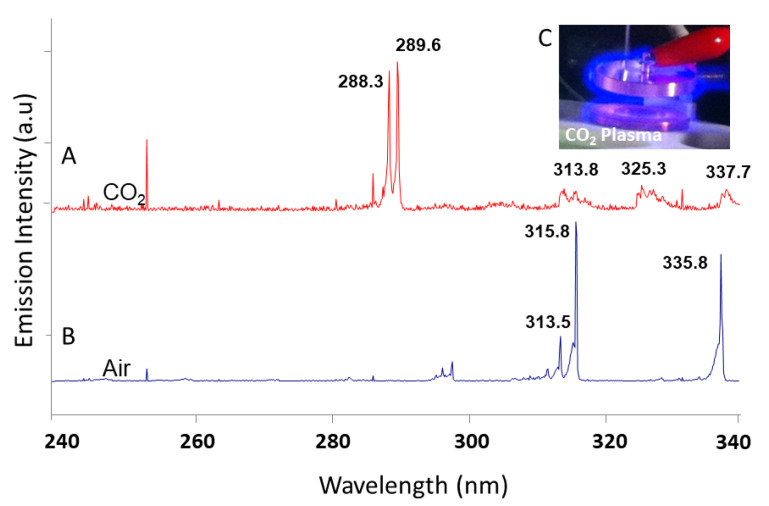
Emission spectra of CO_2_ (**A**) and air (**B**) under low gas pressures (300 Pa); photographs of plasma excited between two plate electrodes (**C**).

The reactive species can be recorded and identified by the emission spectra of CO_2_ glow discharge, which was also monitored during the plasma reactions to assess the capability. Here, CO_2_ and Earth air plasma were generated (CO_2_ plasma, discharge voltage: 1 kV, inset of Figure 7) at 300 Pa using a low-temperature plasma power supply (CTP-2000, Corona Lab, Nanjing, China). Their emission spectra in the UV band (peak position accuracy, 0.14 nm at 313. 2nm) are shown in Figure 7 and were mainly assigned to CO_2_^+^ (288.3, 289.6, 313.8, 325.3, and 337.7 nm) and N_2_ (313.5, 315.8, and 335.8 nm) [32]. These observations provide important experimental evidence that the CO_2_ plasma induced by friction electrostatic discharge in Mars dust storms/devils has powerful oxidizability, which promotes the chemical evolution of surface materials. Material evolution is driven by plasma reactions due to the presence of free ions, UV radiation, free radicals, and chemically reactive neutral species. A new formation mechanism of Martian perchlorates by ESD has been proposed based on CO_2_ glow discharge experiments [32].

## 4. Conclusions

In this work, the structure, function, and performance of a newly developed MEC at Shandong University were introduced. The MEC can both regulate Mars-relative environmental parameters (gas components, gas pressure, sample temperature/humidity, and UV radiation dosage) and simultaneously/sequentially perform multiple spectral measurements such as VIS-NIR, Raman, LIBS, and plasma optical emission spectra.

Overall, three sets of experiments were undertaken to demonstrate the function and performance of MEC and the coupled spectral units. The first set was to investigate the gas pressure, temperature, and humidity functions of chamber. The results show that in the MEC it is easy to control the environmental conditions with good accuracy and repeatability. The wider parameter range and more accurate parametric control make the MEC highly adequate for mineral phase transformation and spectral analysis research work relevant to Mars. The second set of experiments investigated the working performance of the spectral sensors. The spectral characteristics (VIS-NIR, Raman, and LIBS) of some geological samples can be obtained within a few seconds under given Martian environmental conditions. Although it is challenging to record and analyze the natural rocks or heterogeneous samples on the same sampling point, the in situ multiple spectral sensors were designed to have the ability to analyze homogeneous geological samples using hyphenated and fusion spectral analysis methods. This complementary elemental and molecular information from VIS-NIR, Raman, and LIBS spectrometers under simulated harsh Martian conditions will help us better analyze and identify minerals. The third set of experiments investigated the working performance of the glow discharge unit and the emission lines of CO_2_ glow discharge plasma. CO_2_ glow discharge allows us to study chemical material evolution by Martian atmosphere plasma reactions by simulating the electrostatic discharge of Martian dust storm events. The CO_2_ glow discharge plasma was generated and its emission spectra were recorded and assigned. All the tested units of the MEC work well with good accuracy and repeatability.

This MEC and the multiple coupled in situ spectral sensors establish a key link between laboratory experiments and the interpretation of spectral data obtained by the Mars mission. The MEC will help us build a solid foundation for real Martian spectral data analysis and interpretation.

## Data Availability

Not applicable.
